# Ethylene reduces glucose sensitivity and reverses photosynthetic repression through optimization of glutathione production in salt-stressed wheat (*Triticum aestivum* L.)

**DOI:** 10.1038/s41598-021-92086-2

**Published:** 2021-06-16

**Authors:** Zebus Sehar, Noushina Iqbal, M. Iqbal R. Khan, Asim Masood, Md. Tabish Rehman, Afzal Hussain, Mohamed F. AlAjmi, Altaf Ahmad, Nafees A. Khan

**Affiliations:** 1 Department of Botany, Aligarh Muslim University, Aligarh, 202002 India; 2 Department of Botany, School of Chemical and Life Sciences, Jamia Hamdard, New Delhi, 110062 India; 3 Department of Pharmacognosy, College of Pharmacy, King Saud University, Riyadh, 11451 Kingdom of Saudi Arabia

**Keywords:** Plant sciences, Photosynthesis, Plant hormones

## Abstract

Ethylene plays a crucial role throughout the life cycle of plants under optimal and stressful environments. The present study reports the involvement of exogenously sourced ethylene (as ethephon; 2-chloroethyl phosphonic acid) in the protection of the photosynthetic activity from glucose (Glu) sensitivity through its influence on the antioxidant system for adaptation of wheat (*Triticum aestivum* L.) plants under salt stress. Ten-day-old plants were subjected to control and 100 mM NaCl and treated with 200 µl L^−1^ ethephon on foliage at 20 days after seed sowing individually or in combination with 6% Glu. Plants receiving ethylene exhibited higher growth and photosynthesis through reduced Glu sensitivity in the presence of salt stress. Moreover, ethylene-induced reduced glutathione (GSH) production resulted in increased *psbA* and *psbB* expression to protect PSII activity and photosynthesis under salt stress. The use of buthionine sulfoximine (BSO), GSH biosynthesis inhibitor, substantiated the involvement of ethylene-induced GSH in the reversal of Glu-mediated photosynthetic repression in salt-stressed plants. It was suggested that ethylene increased the utilization of Glu under salt stress through its influence on photosynthetic potential and sink strength and reduced the Glu-mediated repression of photosynthesis.

## Introduction

Ethylene is a signaling molecule that acts as a critical modulator of plant processes at the cellular, molecular and whole plant level^1–3^. It plays a crucial role in many biological processes of plants and is involved in almost every aspect of the plant life cycle from germination to senescence^4–6^. It influences the photosynthetic performance of plants under optimal and stressful environments due to the direct ethylene-mediated changes in ribulose 1, 5 bisphosphate carboxylase/oxygenase (Rubisco) activity and carboxylation efficiency, and indirect effect on stomatal aperture^1,7,8^ or by interacting with other signaling molecules^9,10^. The role of ethylene in the alleviation of stresses, including salt stress, has been reviewed^2,11,12^. Cao et al.^13^ reported that the application of ethylene or 1-aminocyclopropane carboxylic acid (ACC) could improve plant response to high salinity through signaling of ethylene by its receptor and augmenting the expression of reactive oxygen species (ROS) scavenger^14^, and modulating antioxidant system. The expression of *ACS2/ACS6* (1-amino-cyclopropane-1-carboxylic acid synthase, ACS) gene in *Arabidopsis* was promoted by activation of mitogen activated protein kinase (MAPK) cascade under salinity stress^15^. Ethylene-insensitive 2 (*EIN2)*-mediated ethylene signaling regulates the induction of *AtERF3* and *AtERF4*, which conferred salinity tolerance^16^. It was earlier observed that ethylene stimulated the activity of PM H^+^-ATP-ase for the modulation of ion homeostasis and conferred salt stress tolerance^17^. Yang et al.^18^ reported that ethylene response factors (*ERFs*) play an essential role in various biotic and abiotic stress responses. Cao et al.^19^ observed that the overexpression of *OSBIERF3*, a B3 sub-group member in rice, improved salt tolerance in tobacco, while Rong et al.^20^ found that overexpression of *TaERF3* significantly improved salt stress tolerance in the *Triticum aestivum*. Similarly, overexpression of a B3 subgroup *ERF* gene, *JcERF*, improved salt stress tolerance in *Arabidopsis* plants, which upregulated photosynthetic characteristics of plant^21,22^. According to the report of Sun et al.^23^, five *ERF* genes were regulated under salt stress, suggesting the role of ethylene signaling in salt stress tolerance through microarray analysis. Expression of *LchERF* also conferred salt stress tolerance at the time of vegetative growth and seed germination in *Nicotiana tabacum*^24^. Ethylene is contemplated as a stress-responsive hormone with a multifaceted role in improving plant growth and photosynthetic performances under abiotic stress conditions^2,9,25^.

Salt stress is significant abiotic stress that adversely affects the productivity of crops worldwide and is anticipated to exaggerate due to the sessile nature of plants and the changing global climate^26,27^. Approximately 7% of the world’s soil is salt-affected, which included 33% of the irrigated agricultural land worldwide^28^. Moreover, about three hectares of arable land gets adversely affected by soil salinization per minute, which causes a 10% increase in the salt-affected area annually^29^. The plants grown under salt stress show disturbance in nutrients and ion homeostasis, osmotic balance and oxidative stress resulting in the excess formation of ROS^2,30,31^. Soil salinity has been found to influence wheat production significantly by adversely affecting photosynthetic efficiency and growth^32,33^. Salt stress affects the photosynthesis efficiency of plants through its effect on the expression of *psbA* and *psbB* of Photosystem II (PS II)^34^. Photosystem II contains at least eight different proteins that are encoded by plastid genes. It is associated with the electron transport and is one of the four multisubunit complex of chloroplast thylakoid membranes. The PS II 32- kDa and 47-kDa membrane proteins, designated as D1 and CP47 are encoded by the plastid *psbA* and *psbB* genes, respectively^35,36^. Plants initiate mechanisms to withstand salt stress conditions, and there might be ethylene involvement in reducing salt stress. In wheat, it has been observed by Khan et al.^37^ that the variation in salt tolerance occurred because of the difference in ethylene, and the tolerant cultivar had higher ethylene evolution than the sensitive cultivar. Moreover, soluble sugars act as a primary messenger that regulates signals for the expression of genes involved in regulating photosynthesis, growth, and metabolism^38^. It has been shown that sugars (such as glucose, fructose and trehalose), based on the concentration, act as a growth regulator and impact photosynthesis and reserve mobilization^39,40^. A low concentration of Glu has been found to stimulate growth, while a high concentration reduced the growth through inhibition in the photosynthetic characteristic of plants. A high concentration of Glu has been reported in salt stress condition in order to protect and alleviate salt stress effects on plants^41,42^. Photosystem II is also susceptible to salt stress and genes that encode the major extrinsic proteins of PSII (*PsbA, PsbB, PsbC*, and *PsbD*) were highly upregulated after melatonin treatment under salt stress, influencing photosynthesis positively both under control and salt stress^43^. Ethylene precursor ACC was found to overcome 6% Glu-mediated development arrest in *Arabidopsis*^44^. The protective mechanisms of the antioxidant system and Glu signaling are triggered through the action of phytohormones by controlling the interactions between plants and environments in response to salinity stress^30,45^. We have earlier reported that ethylene supplementation upregulates the activity of enzymatic and non-enzymatic antioxidants, such as superoxide dismutase (SOD), catalase (CAT), ascorbate peroxidase (APX), glutathione reductase (GR) and reduced glutathione (GSH)^2,25,31,41,45–47^, which work as the primary line of defense in the regulation of abiotic stress factors. Among enzymatic antioxidants, GR controls the production of GSH for the optimal metabolic activities^31,45,48,49^. Ethylene could induce salt tolerance by regulation of GSH^50^. Rehman et al.^51^ found that exogenously applied GSH either alone or in combination with an organic bio-stimulant potentially reduced the deleterious effects of salinity in wheat. Khan et al.^52^ have shown the relationship between ethylene, GSH and sulfur for cadmium tolerance in wheat. In wheat, El-Bassiouny and Bekheta^53^ reported that salt tolerance in tolerant wheat was associated with a lesser reduction in relative water content and lower lipid peroxidation, increased spermidine and spermine and proline accumulation in association with increased ethylene.

Individual studies emphasizing the influence of salt stress on Glu, GSH and PS II have been carried out, but studies on the protection of Glu-mediated inhibition of PS II activity and photosynthetic repression with the involvement of GSH in wheat have not been done.

There is a gap in understanding the mechanisms of how ethylene regulates the photosynthetic activity of plants under salt stress and reduce Glu sensitivity. It was postulated that ethylene would increase the utilization of Glu under salt stress through its influence on photosynthetic potential and sink strength and will reduce the Glu-mediated repression of photosynthesis. Moreover, ethylene will optimize GSH production by influencing ascorbate–glutathione cycle for proper cellular functioning under salt stress. Thus, the reported research was undertaken to test the efficacy of exogenously sourced ethylene (as ethephon, source of ethylene) in protecting the photosynthetic activity, and to evaluate its relation with Glu sensitivity and GSH production for adaptation of wheat grown under salt stress. The involvement of GSH was verified by the use of buthionine sulfoximine (BSO). Buthionine sulfoximine depletes intracellular GSH and induces oxidative stress by specifically and irreversibly inhibiting γ-glutamylcysteine synthetase (γ-GCS), the rate-limiting enzyme of GSH synthesis^54^.

## Results

### Influence of ethephon application on growth and photosynthesis in the presence or absence of Glu

To investigate the effects of ethylene on alleviation of 6% Glu-inhibited growth and photosynthesis, wheat plants were grown with or without 100 mM NaCl (Figure S1). Compared to the control plants, leaf area and plant dry mass decreased by 39.4% and 41.8% in salt grown plants. The individual application of ethephon and Glu had distinct responses. Ethephon application increased leaf area and plant dry mass by 25.1% and 86.7%, while Glu inhibited these parameters by 11.8% and 20.4%, compared to the control plants. Ethephon supplementation reduced glucose sensitivity and promoted leaf area and plant dry mass by 12.4% and 45.9% of plants treated with Glu in the absence of salt, and 19.9% and 36.7% in the presence of salt compared to the control plants (Table 1).

**Table 1:** Chlorophyll content (SPAD value), net photosynthesis (µmol CO_2_ m^−2^ s^−1^), stomatal conductance (mmol CO_2_ m^−2^ s^−1^), intercellular CO_2_ concentration (µmol CO_2_ mol^−1^), leaf area (cm^2^ plant^−1^) and plant dry mass (g plant^−1^) of wheat (*Triticum aestivum* L.) cv. WH 711 treated with 200 µL L^−1^ ethephon (Eth) and / or 6% glucose (Glu) in presence or absence of 100 mM NaCl at 30 d after sowing. Data are presented as treatment mean ± SE (n = 4). Data followed by same letter are not significantly different by LSD test at *p* < 0.05.

Treatment	Chlorophyll content	Net photosynthesis	Stomatal Conductance	Intercellular CO_2_ concentration	Leaf area	Plant dry mass
Control	29.7 ± 1.60^c^	12.4 ± 1.05^de^	342 ± 19.63^ cd^	210 ± 10.11^ cd^	32.2 ± 1.78^bc^	0.98 ± 0.05^de^
NaCl	18.6 ± 1.12^d^	7.8 ± 0.55^f﻿^	272 ± 14.57^e^	141 ± 7.01^e^	19.5 ± 1.41^d^	0.57 ± 0.04^f^.
Eth	46.3 ± 2.11^a^	19.6 ± 1.04^a^	464 ± 18.64^a^	332 ± 9.73^a^	40.3 ± 2.11^a^	1.83 ± 0.11^a^
Glu	25.8 ± 1.64^c^	10.3 ± 0.72^ef^	310 ± 18.50^de^	192 ± 11.93^d^	28.4 ± 1.67^c^	0.78 ± 0.05^ef^
Eth + NaCl	36.5 ± 1.76^b^	14.8 ± 1.10^ cd^	379 ± 20.31^bc^	237 ± 13.49^c^	35.8 ± 1.81^ab^	1.17 ± 0.06^ cd^
Eth + Glu	37.7 ± 1.87^b^	16.4 ± 1.10^bc^	389 ± 22.31^bc^	278 ± 19.13^b^	36.2 ± 1.76^ab^	1.43 ± 0.08^bc^
Eth + Glu + NaCl	42.7 ± 1.98^ab^	18.5 ± 1.04^ab^	403 ± 20.90^b^	308 ± 16.82^ab^	38.6 ± 1.96^a^	1.34 ± 0.07^b^

Salt stress or Glu treatment inhibited photosynthesis in comparison to the control. We studied the effects of ethephon in reversing these inhibitory effects on photosynthesis as ethylene has been found to promote photosynthesis. The photosynthetic parameters, chlorophyll content, net photosynthesis, stomatal conductance and intercellular CO_2_ concentration decreased in salt stressed plants compared to the control plants due to increased Glu sensitivity. Plants receiving ethephon had these parameters higher by 55.8%, 58.1%, 35.6% and 58.1%, respectively compared to the control plants, whereas individual application of Glu increased Glu sensitivity and decreased chlorophyll content by 13.1%, net photosynthesis by 16.9%, stomatal conductance by 9.3% and intercellular CO_2_ concentration by 8.6% compared to the control plants. The reduction in Glu sensitivity was observed in plants which received ethephon with Glu, and these parameters increased by 26.9%, 32.2%, 13.7% and 32.3%, respectively, compared to the control plants. However, ethephon supplementation to plants grown under salt stress resulted in maximally reduced salt-induced Glu sensitivity and increased chlorophyll content by 43.7%, net photosynthesis by 49.2%, stomatal conductance by 17.8% and intercellular CO_2_ concentration by 46.6%, compared to the control plants (Table 1).

### Influence of ethephon or/and Glu on stomatal movement and chloroplast ultrastructural studies

**Figure 1:**
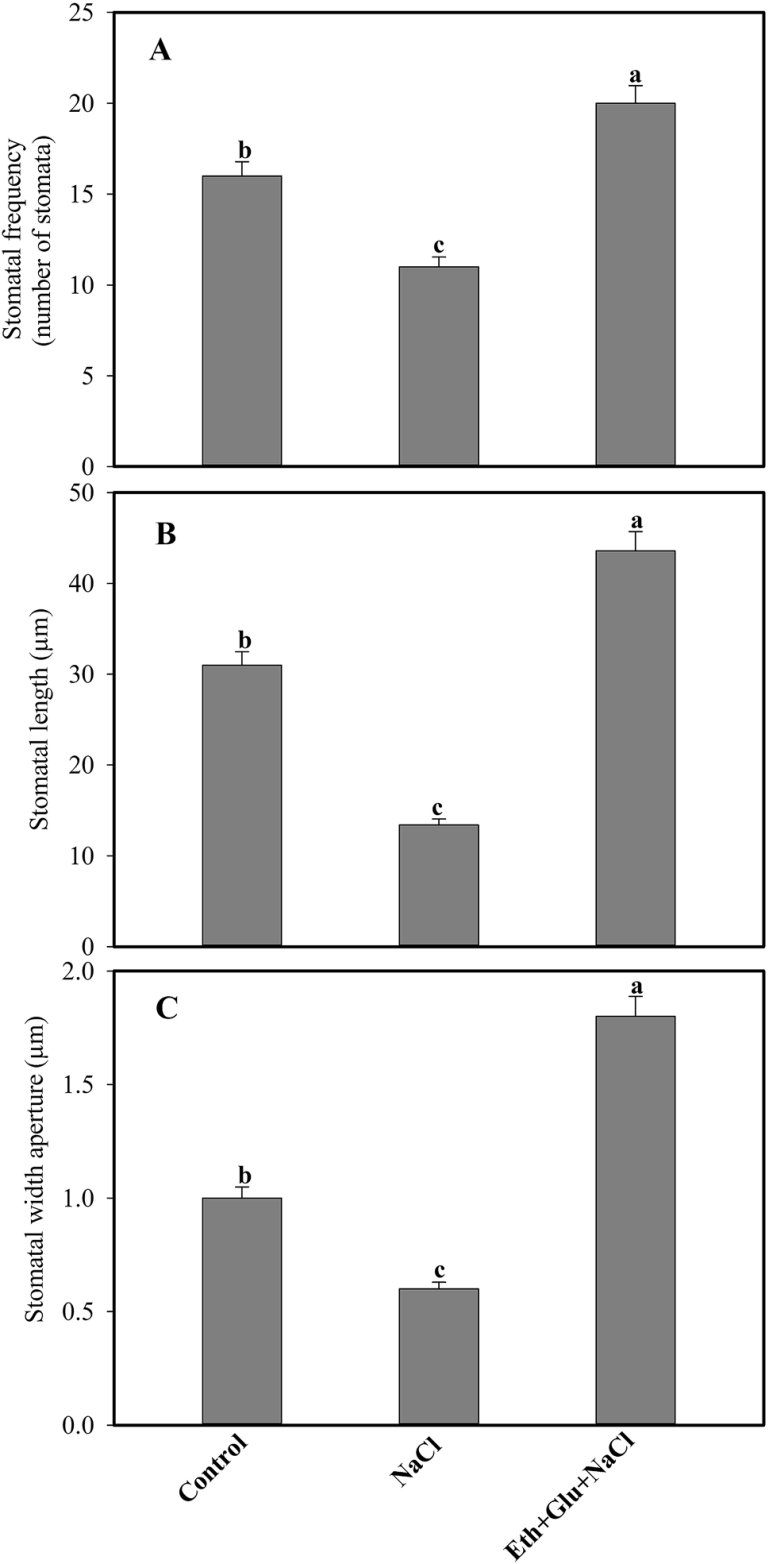
Stomatal frequency (**A**), stomatal length (**B**) and stomatal width aperture (**C**) in wheat (*Triticum aestivum* L.) leaves observed under control, 100 mM NaCl and 200 µL L^−1^ ethephon and 6% Glu with 100 mM NaCl at 30 d after sowing. Data are presented as a treatment mean ± SE (n = 4). Data followed by same letter are not significantly different by LSD test at *p* < 0.05. Eth, ethephon; Glu, glucose.

Stomatal analysis showed that salt treatment induced partial stomatal closure, whereas supplementation of ethephon in the presence of Glu resulted in an increase in the frequency of stomata by 23.1% and 45.4%, compared to the control and salt-treated plants. Salt treatment decreased length and width of stomata by 56.7% and 66.6% compared to the control plants. Ethephon treatment increased stomatal frequency by 23.1% by reducing Glu sensitivity and increased length and width by 40.6% and 80.1% in the presence of Glu and salt stress compared to the control plants (Fig. 1).

Under control conditions, plants had a well-developed thylakoid system, but it disorganized under salt stress. However, plants that received ethephon had positive effects on chloroplast and thylakoid system even when they were treated with Glu and salt (Figure S2). Plants treated with salt stress exhibited partially closed stomata compared to the control plants. In contrast, plants receiving ethephon and Glu in presence of salt had more opened stomata compared to salt grown plants.

### Effect of ethephon or/and Glu on PSII activity and *psbA* and *psbB* expression

We examined PSII activity to determine the effects of ethylene on photosynthesis of plants in the presence of Glu and salt stress. Plants grown with 100 mM NaCl exhibited decreased actual PSII efficiency by 17%, maximal PSII efficiency by 21.0%, intrinsic PSII efficiency by 12.5%, electron transport rate by 15.7%, photochemical quenching by 17.2%, whereas non-photochemical quenching increased by 27.0% compared to the control plants. Supplementation of ethephon decreased Glu sensitivity and improved the above parameters compared to the control and salt-grown plants. In contrast, individual application of Glu decreased the above characteristics by increasing Glu sensitivity as compared to the control plants, with increased NPQ compared to the control plants. Follow up treatment of ethephon to Glu-treated plants improved the above characteristics by reducing the Glu sensitivity compared to the control plants. In the presence of Glu, application of ethephon on salt grown plants maximally decreased Glu sensitivity. It increased actual PSII efficiency by 23.7%, maximal PSII efficiency by 21%, intrinsic PSII efficiency by 23.6%, electron transport rate by 19.6%, photochemical quenching by 24.1%, respectively compared to the control plants. In contrast, NPQ decreased by 1.4%, compared to the control plants (Table 2).

**Table 2:** Actual PSII efficiency, maximal PSII efficiency, intrinsic PSII efficiency, photochemical quenching (qP), non-photochemical quenching (NPQ) and electron transport rate of wheat (*Triticum aestivum* L.) cultivar WH 711 treated with 200 µL L^−1^ ethephon (Eth) and / or 6% glucose (Glu) in presence or absence of 100 mM NaCl at 30 d after sowing. Data are presented as treatment mean ± SE (n = 4). Data followed by same letter are not significantly different by LSD test at *p* < 0.05.

Treatment	Actual PSII efficiency	Maximal PSII efficiency	Intrinsic PSII efficiency	qP	NPQ	Electron transport rate
Control	0.59 ± 0.03^bc^	0.81 ± 0.04^bc^	0.72 ± 0.04^bc^	0.87 ± 0.043^bc^	0.69 ± 0.034^c^	183.6 ± 9.27^bc^
NaCl	0.49 ± 0.02^d^	0.64 ± 0.03^d^	0.63 ± 0.03^d^	0.72 ± 0.036^d^	0.87 ± 0.043^a^	154.7 ± 8.23^d^
Eth	0.64 ± 0.03^ab^	0.91 ± 0.60^ab^	0.84 ± 0.04^ab^	0.96 ± 0.048^b^	0.63 ± 0.031^ cd^	223.7 ± 11.26^a^
Glu	0.52 ± 0.02^ cd^	0.69 ± 0.30^ cd^	0.69 ± 0.03^ cd^	0.76 ± 0.038^ cd^	0.73 ± 0.036^b^	166.8 ± 8.47^ cd^
Eth + NaCl	0.56 ± 0.03^b^	0.79 ± 0.80^b^	0.68 ± 0.03^c^	0.88 ± 0.040^c^	0.76 ± 0.038^ab^	203.4 ± 10.45^b^
Eth + Glu	0.61 ± 0.03^ab^	0.86 ± 0.60^ab^	0.78 ± 0.04^ab^	0.94 ± 0.047^ab^	0.79 ± 0.043^b^	214.4 ± 10.9^ab^
Eth + Glu + NaCl	0.73 ± 0.036^a^	0.98 ± 0.09^a^	0.89 ± 0.04^a^	1.08 ± 0.054^a^	0.68 ± 0.036^c^	219.7 ± 11.1^ab^

**Figure 2:**
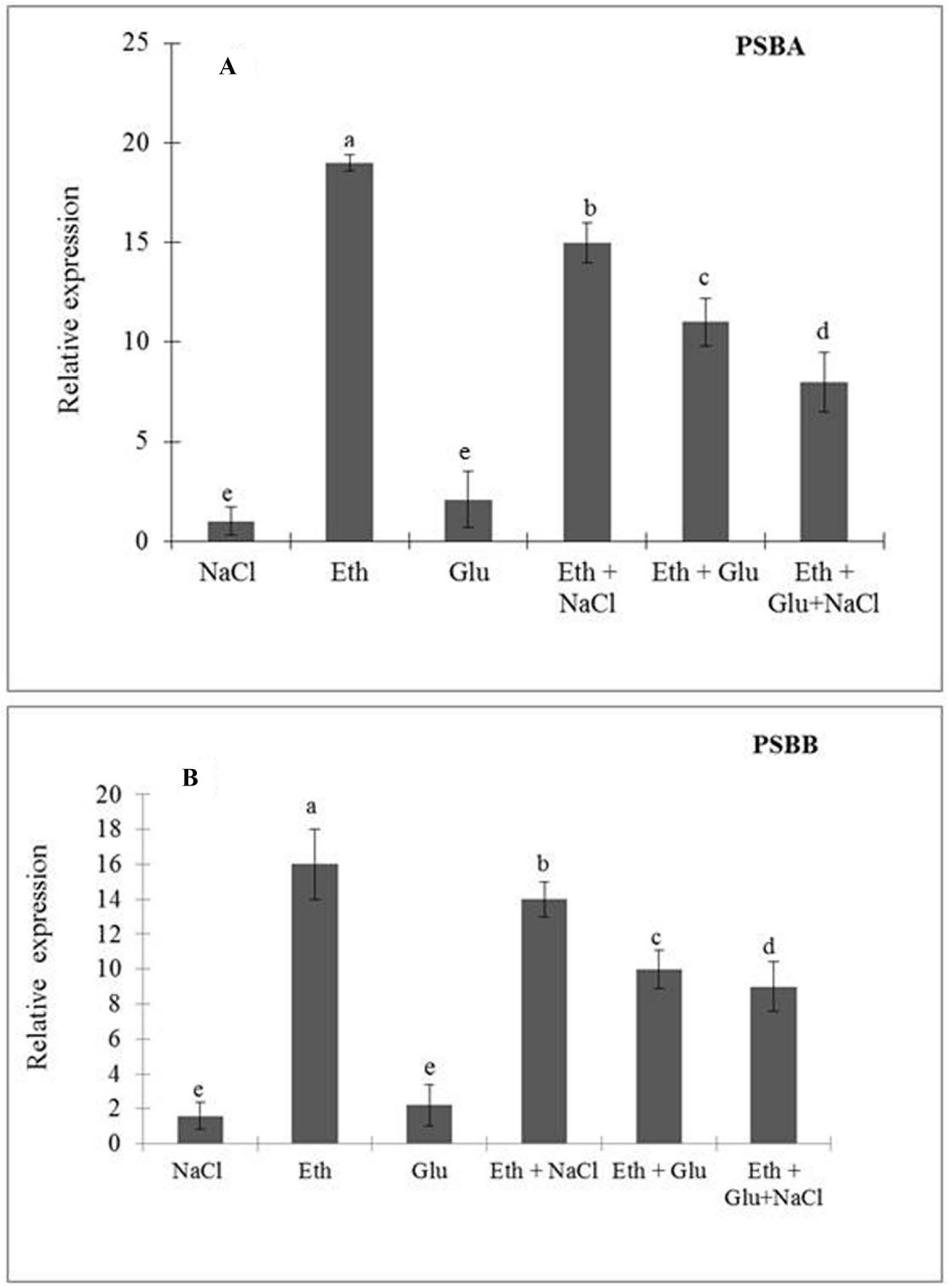
Relative gene expression of *psbA* (**A**) and *psbB* (**B**) in wheat (*Triticum aestivum* L.) induced by 200 µL L^−1^ ethephon in presence or absence of 6% Glu and 100 mM NaCl at 30 d after sowing. Expression fold as compared to control was calculated using ΔΔCt method. Actin gene was used as a reference gene. Data are presented as treatment mean ± SE (n = 4). Data followed by same letter are not significantly different by LSD test at *p* < 0.05. Eth, ethephon; Glu, glucose.

The expression levels of *psbA* and *psbB* enhanced significantly by ethephon treatment alone or in the presence of NaCl/Glu due to reduced Glu sensitivity compared with the control. NaCl treatment alone slightly increased the level of the two genes over control, but the increase was non-significant. Among the various treatments, the maximum increase in the expression of these genes was observed with the treatment of ethephon alone (Fig. 2).

### Influence of ethephon on oxidative stress and antioxidant system in presence or absence of Glu under salt stress

Plants receiving NaCl or/and Glu exhibited higher content of H_2_O_2_ and TBARS due to the increased salt and Glu induced oxidative stress in comparison to the control plants. Individual application of ethephon reduced the content of H_2_O_2_ and TBARS compared to the control plants. Under salt stress condition, ethephon application reduced the content of H_2_O_2_ and TBARS by 43.6% and 54.1%, respectively, compared to the salt-treated plants. Application of ethephon reversed the inhibitory effect of Glu by decreasing Glu sensitivity and reduced the content of H_2_O_2_ and TBARS by 9.4% and 23.8%, respectively, compared to the control plants. However, the application of ethephon along with Glu under salt stress maximally reduced Glu sensitivity and decreased the content of H_2_O_2_ and TBARS by 20.9% and 34.3%, respectively, compared to the control plants (Table 3).

**Table 3:** H_2_O_2_ content (nmol g^−1^ FW), content of thiobarbituric acid reactive substances (TBARS, nmol g^−1^ FW), activity of superoxide dismutase (SOD, U mg^−1^ protein min^−1^), ascorbate peroxidase (APX, U mg^−1^ protein min^−1^), glutathione reductase (GR, U mg^−1^ protein min^−1^) and content of reduced glutathione (GSH, nmol g^−1^ FW) of wheat (*Triticum aestivum* L.) cultivar WH 711 grown with/ without 100 mM NaCl and 200 µL L^−1^ ethephon and / or 6% glucose (Glu) at 30 d after sowing. Data are presented as treatment mean ± SE (n = 4). Data followed by same letter are not significantly different by LSD test at *p* < 0.05.

Treatment	H_2_O_2_ content	TBARS content	SOD activity	APX activity	GR activity	GSH content
Control	19.1 ± 1.27^c^	6.7 ± 0.46^c^	7.63 ± 0.43^d^	2.11 ± 0.16^d^	2.38 ± 0.15^d^	266 ± 15.7^c^
NaCl	34.4 ± 1.98^a^	12.2 ± 0.62^a^	11.2 ± 0.73^c^	3.73 ± 0.21^c^	3.68 ± 0.23^c^	309 ± 16.1^bc^
Eth	13.7 ± 1.47^d^	5.5 ± 0.35^ cd^	12.9 ± 0.78^c^	4.1 ± 0.27^c^	3.74 ± 0.26^c^	319 ± 17.9^bc^
Glu	24.5 ± 1.53^b^	10.1 ± 0.62^﻿b^	12.14 ± 0.83^c^	3.21 ± 1.64^c^	3.71 ± 0.24^c^	315 ± 14.6^bc^
Eth + NaCl	19.4 ± 1.13^c^	5.6 ± 0.33^ cd^	16.9 ± 0.86^b^	7.07 ± 0.88^b^	4.69 ± 0.29^b^	348 ± 20.6^b^
Eth + Glu	17.3 ± 1.01^ cd^	5.1 ± 0.32^d^	17.6 ± 0.91^b^	7.64 ± 0.54^b^	5.36 ± 0.31^b^	356 ± 20.06^b^
Eth + Glu + NaCl	15.1 ± 0.81^d^	4.4 ± 0.26^d^	21.6 ± 1.25^a^	9.42 ± 0.70^a^	6.24 ± 0.38^a^	462 ± 27.9^a^

In the presence of ethephon, plants treated with Glu and grown under salt stress showed maximum activity of the antioxidant enzymes. Application of Glu resulted in increased activity of SOD, APX and GR by 59.1%, 52.1% and 55.9%, respectively, compared to the control plants. Plants receiving ethephon in the presence of Glu upregulated the activity of SOD, APX and GR by 130.6%, 262.1% and 125.2%, compared to the control plants. However, ethephon application maximally increased the activity of SOD, APX and GR by 183.1%, 346.4% and 162.2% in the presence of Glu and salt compared to the control plants (Table 3).

Salt stress significantly increased GSH content as compared to the control plants. Individual application of ethephon and Glu increased GSH content by 1.2-times equally compared to the control plants. Supplementation of ethephon increased GSH content by 1.33-times when Glu was present compared to the control plants, whereas in the presence of Glu and salt, ethephon application maximally increased GSH content by 1.73-times compared to the control plants (Table 3).

### Effect of ethephon or/and Glu on ethylene evolution

**Figure 3:**
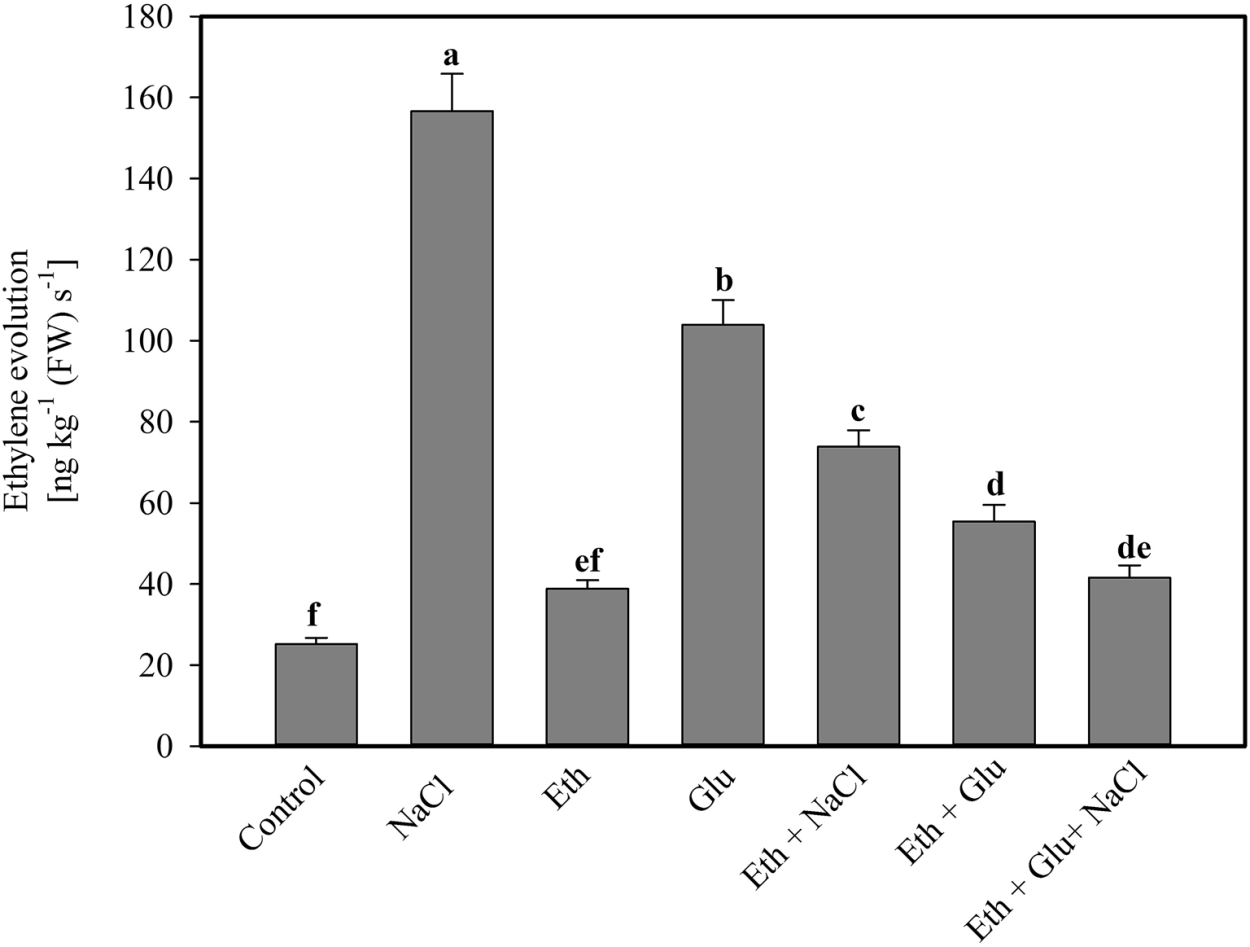
Ethylene evolution in wheat leaves treated with 200 µL L^−1^ ethephon and / or 6% Glu in the presence or absence of 100 mM NaCl at 30 d after sowing. Data are presented as treatment mean ± SE (n = 4). Data followed by the same letter are not significantly different by LSD test at *p* < 0.05. Eth, ethephon; Glu, glucose.

Plants grown with 100 mM NaCl exhibited increased ethylene evolution as compared to the control plants. Salt stress increased ethylene evolution by 6.1-times compared to the control plants. The individual application of ethephon and Glu increased ethylene evolution compared to the control plants and decreased compared to the salt-treated plants. However, ethephon application reversed the effects of Glu and decreased ethylene evolution compared to the Glu-treated plants. Ethephon also reversed the effects of NaCl and decreased ethylene evolution by 2.12-times compared to the salt-treated plant. The supplementation of ethephon to plants treated with Glu under salt stress resulted in a maximal and equal decrease in ethylene evolution by 2.5-times as compared to the Glu-treated plants (Fig. 3).

### Effect of glutathione synthesis inhibitor on ethylene-mediated alleviation

Supplementation of ethephon lowered the impact of salt stress on photosynthetic parameters via an increase in GSH production. The ethylene-mediated alleviation of salt stress through enhanced GSH production was not observed when plants received glutathione synthesis inhibitor, BSO. Supplementation of BSO to salt-treated plants decreased GSH content with the highest decrease in photosynthetic and growth performance compared to the other treatments. Plants treated with ethephon along with Glu in the presence of NaCl and BSO exhibited a lesser decrease in photosynthetic and growth characteristics of the plant compared to the plants treated with ethephon along with NaCl and BSO (Table 4).

**Table 4:** Chlorophyll content (SPAD value), net photosynthesis (µmol CO_2_ m^−2^ s^−1^), content of reduced glutathione (GSH, nmol g^−1^ FW), leaf area (cm^2^ plant^−1^) and plant dry mass (g plant^−1^) of wheat (*Triticum aestivum* L.) cultivar WH 711 treated with 200 µL L^−1^ ethephon (Eth) and / or 6% glucose (Glu) and 0.5 mM buthionine sulfoximine (BSO) at 30 d after sowing. Data are presented as treatment mean ± SE (n = 4). Data followed by the same letter are not significantly different by LSD test at *p* < 0.05.

Treatment	Chlorophyll content	Net photosynthesis	GSH content	Leaf area	Plant dry mass
Control	30.8 ± 1.7^c^	12.3 ± 1.1^bc^	252 ± 14.1^d^	31.8 ± 1.8^b^	0.96 ± 0.04^c^
NaCl	19.8 ± 1.2^e^	8.4 ± 0.8^ cd^	298 ± 15.6^c^	19.4 ± 1.4^d^	0.67 ± 0.04^e^
Eth + NaCl	38.4 ± 2.2b	15.6 ± 1.1^b^	329 ± 18.2^b^	36.8 ± 2.1^ab^	1.21 ± 0.05^b^
Eth + NaCl + BSO	12.6 ± 0.63^d^	5.9 ± 0.29^c^	22 ± 2.6^﻿f^	11.8 ± 0.59^c^	0.43 ± 0.021^d^
Eth + Glu + NaCl	44.2 ± 2.8^a^	19.4 ± 1.3^a^	462 ± 25.8^a^	39.2 ± 2.8^a^	1.37 ± 0.10^a^
Eth + Glu + NaCl + BSO	14.9 ± 0.74^bc^	7.2 ± 0.36^ab^	30 ± 2.9^e^	14.6 ± 0.73^ab^	0.56 ± 0.028^ab^

## Discussion

Salt stress is one of the most commonly encountered environmental stresses limiting plant growth and productivity^13,41^. Ethylene's involvement in regulating various abiotic stress responses, including salt stress, has been studied, but the present study revealed a sustainable approach to overcome Glu-inhibited photosynthetic and growth of salt grown wheat plants by ethylene.

Studies show that salt stress induces excess ethylene formation and inhibition of photosynthetic processes^13,41,55^. The present research has shown that ethylene influences defense system of plants and reduced Glu sensitivity to alleviate the salt stress-inhibited photosynthesis. The excess ethylene produced under salt stress was minimized on ethephon application to optimal range, which favourably regulated GSH production via a control on the activity of enzymes of the ascorbate–glutathione cycle. The higher reduced state developed due to ethylene-induced GSH production protected and improved photosynthetic performances and growth of plants under salt stress. Supplementation of ethylene increased the photosynthetic performance of plants via increase in stomatal conductance and stomatal behaviour, which allowed more flux of intercellular CO_2_ for fixation. It has been earlier shown that exogenously applied ethylene increases photosynthetic performance in *B. juncea* through an increase in stomatal conductance^1,2^ and increased diffusion rate of CO_2_ through intercellular spaces and stomatal aperture^2,56^. The higher photosynthetic rate, together with increased photosynthesizing area, resulted in increased plant dry mass. The ethylene mediated higher growth also resulted in reduced Glu sensitivity through its utilization by the growing plants and reversed the feedback inhibition. Earlier, it has been reported by Khan et al.^2^ that ethylene was involved in increased leaf area and plant dry mass in Cd-treated plants.

Photosystem II is a highly oligomeric protein complex that requires some mechanisms for control and protection against abiotic stress. Minai et al.^57^ reported that elicitors help in the protection of PSII by expression of the constitutive sub-units. Application of ethylene resulted in the reversal of photo-inhibition and reduced damage to photosynthetic attributes caused by salinity stress. In the present report, ethylene induced the expression of *psbA* and *psbB*, and maintained higher GSH level of the cell that accounted for the increased PSII activity and prevented the PSII assembly from salt induced damage. On a similar line, application of spermidine has been shown to enhance expression of *psbA* and *psbB* genes in tall fescue plants under heat stress^35^. Kim et al.^58^ also reported that ethylene regulated hypocotyl growth in *Arabidopsis* plant through involvement of gene expression of PSII, whereas abscisic acid (ABA) treatment regulated gene expression of *psbA* under drought stress in wheat plants. However, report on the ethylene-induced expression of PSII genes and the integrity and efficiency of PSII to restore photosynthetic attributes of plants with enhanced Glu sensitivity under salt stress has not been shown. Application of ethylene reduced Glu sensitivity under salt stress by its increased utilization to meet the growing demands of the plants, and increased the efficiency of PSII and electron transport rate, which limited the production of singlet oxygen under salt stress. We have shown earlier that exogenously applied ethylene increased PSII activity by limiting metals-induced stress ethylene to optimal ethylene level in *B. juncea*^2,46^. The supplementation of ethylene was also shown to stimulate the activity of various antioxidant enzymes and growth and photosynthetic performances^2,31,59^. However, there is no report available regarding the role of ethylene in Glu-inhibited photosynthetic performances involving PSII activity in wheat under salt stress.

It was hypothesized that there could be some changes in the guard cell induced by ethylene which led to the opening of stomata even in the presence of Glu and salt. It has been earlier reported that stomatal movement is controlled by influx/efflux of Ca^2+^ ion and also depends on ABA concentration of the guard cells^45,60^. The present study also confirms the relationship between stomatal behaviour and ethylene evolution. It may be said that there is ethylene signaling in guard cells to induce changes in abscisic acid (ABA) level for stomatal movement in the presence of Glu under salt stress. Fatma et al.^61^ showed ethylene in the presence of sulphur induced ABA changes in guard cell, leading to increased stomatal conductance and structure and efficiency of photosynthetic apparatus in *Brassica juncea* under salt stress. The application of ethylene increased GSH content that led to cellular redox homeostasis and controlled stomatal movement.

Additionally, ethylene also modulated the activity of antioxidant enzymes to protect the plants from oxidative damage. It is clear from the above report that there is a strong correlation between ethylene evolution, GSH content and stomatal movement. The observed well-organized chloroplast structure and well-arranged thylakoid system under the influence of ethylene were due to the increased activity of antioxidant enzymes and GSH production and decreased level of lipid peroxidation. As a result, the light-harvesting complex proteins were stabilized in the well-organized chloroplast that improved the photosynthesis under salt stress. Ma et al.^62^ found that wheat mutant lines with decreased ethylene sensitivity exhibited enhanced salt tolerance, suggesting ethylene sensitivity relation to salt tolerance. Overexpression of *TaERF3* (an ethylene-response factor) in wheat resulted in increased tolerance to salt and drought stress^63^. The *ERF* works by reduction of H_2_O_2_ content and enhancement of photosynthetic capabilities, osmolyte accumulation and thus tolerance to salt, drought, and cold stresses^63,64^. The maintenance of an efficient photosynthetic system under stress conditions is one of the main issues for plants to attain their required energy. Khan et al.^52^ reported that ethylene was responsible for improving Cd tolerance by enhanced GSH synthesis with selenium and sulfur availability.

In the presence of Glu, the content of H_2_O_2_ and TBARS increased which was suppressed upon ethylene application. Ethylene enhanced the activity of enzymes of the ascorbate–glutathione cycle and produced higher GSH content that limited the oxidative stress generated by Glu and salt together. This report agrees with earlier studies in which application of ethylene reduced oxidative stress and increased the activity of antioxidant enzymes in *B. juncea*^2,46,65^. It has been shown that Cd stress increased ACS (1-aminocyclopropanecarboxylic acid synthase) activity and ethylene evolution, whereas ethylene supplementation optimized ACS activity and ethylene evolution in presence of Cd and stimulated GSH production in *B. juncea*^2^. It has been earlier reported that the GSH is synthesized through an immediate precursor by sulfur-containing amino acids such as cysteine that serves as a metabolite for ethylene formation through S-adenosylmethionine^41^. The present study reported that optimal ethylene promoted GSH production and helped in maintaining the redox status of the cell. Supplementation of 150 mM NaCl increased ethylene evolution in nine cultivars of lactuca seeds^66^. Under salt stress condition, *MPK6*, a key regulator of ethylene biosynthesis is necessary for salt acclimation^67^. In the present study, it was observed that application of ethylene decreased Glu sensitivity and stress ethylene evolution, together with the increased antioxidant enzyme activities and GSH and growth and photosynthetic performances under salt stress.

The reversal of Glu sensitivity-mediated inhibition of photosynthetic and growth performance of plants under salt stress by ethylene was through regulation on GSH production. The use of GSH biosynthesis inhibitor, BSO reversed the effects of ethylene in protection of photosynthesis and growth because of depleted levels of GSH. This depletion in GSH production exasperated oxidative damage through an increase in H_2_O_2_ and TBARS content, which consequently reduced photosynthetic and growth characteristics. The importance of GSH in potentially reducing the deleterious effects of salinity^51^, and the relationship of GSH and ethylene in Cd tolerance^52^ in wheat has been shown.

Based on our study, it is concluded that there is higher accumulation of Glu and stress ethylene formation under salt stress condition, causing reduction in photosynthetic performance of wheat plants. The ethylene supplementation through ethephon optimized the level of Glu and reduced its inhibitory effect on the photosynthetic performance. This increase in the photosynthetic performance was due to optimization of stress ethylene under salt stress by the supplementation of ethylene and through regulation of GSH production, which enabled decreased ROS production. These findings were substantiated with the use of GSH biosynthesis inhibitor (BSO). This confirmed the role of ethylene in reversal of Glu-inhibited photosynthetic performance under salt stress via ethylene-regulated GSH production. The present study suggests that ethephon supplementation in an agricultural system may be adopted for increased photosynthesis and growth under salt stress.

## Materials and methods

### Plant material, growth conditions, and treatments

Healthy uniform seeds of wheat (*Triticum aestivum* L.) cv. WH 711 (winter wheat) which grows up to 100–105 cm. and is of 125–130 days duration and yield up to 45–50 quintals per hectare, under optimum conditions, were taken for the experiment. Seeds were obtained from National Seeds Corporation, New Delhi, India. These seeds were surface sterilized with 0.01% HgCl_2_ followed by washings with double distilled water. The sterilized seeds were sown in 23-cm diameter earthen pots filled with acid-washed sand, as described earlier^41^. Plants in each pot were supplemented with 300 mL Hoagland nutrient solution on alternate days, and 100 mM NaCl was added at ten days after sowing (DAS) and then again at 20 DAS (at an interval of 10 days). Plants at the age of 20 DAS were treated with 200 µL L^−1^ ethephon (2-chloroethyl phosphonic acid; an ethylene releasing compound) on foliage individually or in combination with 6% Glu in the presence or absence of NaCl. The effect of ethephon in reducing Glu sensitivity in the presence of salt was assessed by recording observations on growth and photosynthetic parameters, oxidative stress as the content of H_2_O_2_ and lipid peroxidation. The enzymes of the ascorbate–glutathione pathway and GSH production were also noted. It was suggested that the effects of ethylene in reducing Glu sensitivity and reversing the effects of salt stress-induced oxidative stress were through optimized GSH production. Therefore, the plants grown with salt or salt plus Glu were treated with GSH biosynthesis inhibitor, buthionine sulfoximine. The selection of concentrations 200 µL L^−1^ ethylene and 6% Glu was based on the findings of the earlier experiment^8,41^. The pots were kept in the net house of the Department of Botany, Aligarh Muslim University, Aligarh (India) under natural day/night conditions with photosynthetically active radiation (PAR, 650 µ mol m^−2^ s^−1^; average day/night temperatures of 21 °C /17 °C (± 3 °C), and relative humidity of 68 ± 5%. The observations on different parameters were taken at 30 DAS. In each experiment, treatments were arranged in a complete randomized block design, and the number of sets for each treatment was four (n = 4).

### Determination of growth and photosynthesis

The plants were uprooted gently, washed under running tap water to remove dust and dried in a hot air oven at 80 °C until constant weight for plant dry mass. Leaf area was measured by using a leaf area meter (LA211, Systronic, New Delhi, India).

Net photosynthesis, stomatal conductance and intercellular CO_2_ concentration were measured in fully expanded uppermost intact leaves of plants in each treatment using Infrared Gas Analyzer (CID-340, photosynthesis system, Bioscience, USA). The measurements were done between 11 and 12 h at light saturating intensity, temperature (22 °C) and relative humidity of approximately 60%. Chlorophyll content was measured in intact second top leaves of the plants with the help of SPAD Chlorophyll meter (SPAD, 502 DL PLUS, Spectrum technologies, USA).

### Measurement of chlorophyll fluorescence

The chlorophyll fluorescence measurements were made using Junior-PAM chlorophyll fluorometer (Heinz Walz, Germany). The details of the procedure are given in Supplementary File S1.

### Stomatal and chloroplast ultrastructural studies

Scanning electron microscopy (SEM) of leaf samples was done by following the procedure described earlier^45^. Fresh leaf samples were taken from the axillary positions (leaves with 1.5 × 1.5 cm in size) and were preferably air-dried in desiccators. The desiccator-dried leaf samples were first fixed with 2.5% glutaraldehyde and 2% paraformaldehyde in 0.1 M phosphate buffer (pH 7.0) in equal quantity for about 4 h. The fixed leaf samples were washed with phosphate buffer three times for 15 min. The samples were then postfixed with osmium tetra oxide in potassium phosphate buffer (pH 7.0) for 1 h and were subsequently washed three times with the same phosphate buffer for 15 min at each step. The specimens were dehydrated by a graded series of ethanol (50, 70, 80, 90, 95 and 100%) for about 15–20 min at each step and transferred to the mixture of alcohol and isoamyl acetate mixed in an equal ratio for about 30 min. Thereafter, the samples were transferred to pure isoamyl acetate for 1 h. Further, specimens were dehydrated in Carl Zeiss EVO (Germany) scanning electron microscope critical point dryer with liquid CO_2_. Finally, the dehydrated specimens were coated with gold–palladium and observed under Carl Zeiss EVO scanning electron microscope at extra high tension or high voltage at 15 kV and magnification of 150× or 1000×. The stomata were observed under scanning electron microscope at the resolution of 150× and 1000× and stomatal frequency was determined by counting the number of stomata in the microscope field of view.

Leaf tissues for studying chloroplast ultrastructure were prepared for transmission electron microscopy by adopting the method of Sandalio et al.^68^ with slight modifications. Leaf samples were cut with razor blade into 1 mm^2^ segments and fixed in 2.5% glutaraldehyde solution in 50 mM phosphate buffer (pH 6.8) for 2.5 h at room temperature. Leaf tissue was then post-fixed for 30 min in 1% osmium tetroxide in 50 mM sodium cacodylate buffer (pH 7.2) and dehydrated in ethanol graded series (30–100%, v/v). After dehydration in the graded series of ethanol, the tissue was embedded in Spur resin. Ultrathin sections were stained with uranyl acetate and lead citrate and examined using a transmission electron microscope (ZEOL 2100F, USA) high voltage at 120 kV and 6000× magnification. The chloroplast ultrastructure (thylakoid membranes) was observed from transmission electron microscopy images.

### Contents of H_2_O_2_ and TBARS

Leaf H_2_O_2_ was determined by adopting the method of Okuda et al^69^. The status of lipid peroxidation in leaves was estimated by the method described by Dhindsa et al.^70^ as the content of thiobarbituric acid reactive substances (TBARS). The details of the procedure are given Supplementary File S1.

### Assay of activity of antioxidants enzyme and content of reduced glutathione

Fresh leaves were homogenized with an extraction buffer containing 0.05% (v/v) Triton X-100 and 1% (w/v) PVP in potassium-phosphate buffer (100 mM, pH 7.0) using chilled mortar and pestle. The supernatant obtained after centrifugation was used for the assay of SOD (EC; 1.15.1.1) and GR (EC; 1.6.4.2) enzymes. For the assay of APX (EC; 1.11.1.11), 2.0 mM ascorbate was supplemented with extraction buffer. The activity of SOD was assayed by the method of Beyer and Fridovich and Giannopolitis and Ries^71,72^. The activity of APX was determined following the method of Nakano and Asada^73^ by recording the decrease in the absorbance of ascorbate at 290 nm. The activity of GR was determined by the method of Foyer and Halliwell^74^ by monitoring the glutathione-dependent oxidation of NADPH at 340 nm. The details of the method have been given in Supplementary File S1. Reduced glutathione (GSH) was determined following the method of Griffith^75^. The details of the procedure are given in Supplementary File S1.

### RNA isolation, cDNA synthesis and real time RT-PCR

Total RNA was isolated from the leaves of treated and control plants using TRIzol (Ambion, Life Technologies, USA), following the manufacturer’s instructions. Isolated RNA was quantified using Nanodrop spectrophotometer (Thermo Scientific, USA). The integrity of the RNA was determined by running 1 μg RNA of each sample on gel electrophoresis in formaldehyde gels, as described by Turano et al^76^.

One μg total RNA of control and treated samples was used for the first-strand complementary DNA (cDNA) synthesis, using 20 U/μL Molony murine leukaemia virus reverse transcriptase (MuMLV) enzyme (Fermentas, USA) at 42 °C for 50 min and at 70 °C for 10 min. The reverse transcription reaction was carried out using 2.5 μMOligo (dT)18 primer (Fermentas, USA) and 10 mM dNTPs. The cDNA sequences of selected genes were taken from NCBI, and real-time primers were designed by using online primer designing software (IDT) (Supplementary Table S1).

Real-time PCR (RT-PCR) was performed in 96-well reaction plate (Roche, Germany) containing 20 μL reaction mixture of × 10 reaction buffer, 2 mM dNTPs, 1 mM MgCl_2_, 0.35 μM each of forward and reverse primers, 1 μL SYBRgreen (× 10), 10 μgc DNA template and 5 U Taq polymerase on a thermal cycler (Light cycler 480 II, Roche, Germany). All quantifications were normalized to actin DNA fragment amplified by β-actin forward and β-actin reverse primers. The actin gene was used as an internal control for evaluating the efficiency of real-time PCR for particular genes. The reaction conditions for real-time PCR were as follows: 95 °C for 3 min for initial denaturation, followed by 40 cycles of 95 °C (20 s), 66 °C (1 min) and 72 °C (1 min) with 5 min of final extension at 72 °C. The RT-PCR product was resolved on 1.2% agarose gel. The specificity of amplicons was verified by melting curve analysis (60–95 °C) after 40 cycles. All reactions were performed in three biological replicates (with three technical replicates of each), using gene-specific primers and actin primer as an internal control. Primer pairs used for quantitative RT-PCR are listed in Table S1.

The data were taken as the expression of the gene of interest in relation to the internal control in the treated sample compared with the untreated control.

### Estimation of ethylene level

Level of ethylene was estimated using a gas chromatograph. The details of the procedure have been given earlier^41,65^ and presented in the Supplementary File S1.

### Statistical analysis

Data were analysed statistically using analysis of variance (ANOVA) by SPSS 17.0 for windows and presented as mean ± SE (n = 4). The least significant difference was calculated for the significant data at *p* < 0.05. Bars showing the same letter are not significantly different by the least significant difference (LSD) test at *p* < 0.05.

### Ethical statement

We confirm that all methods involving plant seeds were carried out in accordance with relevant guidelines and regulations.

## Supplementary Information

Supplementary Information.
