# Supplementary material for: Ethylene reduces glucose sensitivity and reverses photosynthetic repression through optimization of glutathione production in salt-stressed wheat (Triticum aestivum L.)
Source: Sci Rep. 2021 Jun 16;11:12650. doi: 10.1038/s41598-021-92086-2 (PMC8209215; doi:10.1038/s41598-021-92086-2)
Supplement: Supplementary file 1 — Supplementary Information. [file 41598_2021_92086_MOESM1_ESM.docx]

Ethylene reduces glucose sensitivity and reverses photosynthetic repression through optimization of glutathione production in salt-stressed wheat (*Triticum aestivum* L.)

Zebus Sehar^1^, Noushina Iqbal^2^, M. Iqbal R. Khan^2^, Asim Masood^1^, Afzal Ahmad^3^, Md. Tabish Rehman^3^, M. F. Al-Ajmi^3^, Altaf Ahmad^1,^, Nafees A. Khan^1,*^

^1^Department of Botany, Faculty of Life Sciences, Aligarh Muslim University, Aligarh 202002, India

^2^Department of Botany, School of Chemical and Life Sciences, Jamia Hamdard, New Delhi 110062, India

^3^Department of Pharmacognosy, College of Pharmacy, King Saud University, Riyadh 11451, KSA

***** Correspondence: naf9.amu@gmail.com

**Table S1. Primer pairs used for quantitative RT-PCR**

| **S.No** | **Gene** | | | **Forward primer** | **Tm**  **^◦^ C** | **Length**  **(bp)** | **Reverse primer** | **Tm**  **^◦^ C** | | **Length**  **(bp)** |
| --- | --- | --- | --- | --- | --- | --- | --- | --- | --- | --- |
| 1. | *PSBA* | | | ATATTGTGGCCGCTCAT | 49.9 | 17 | TCCGTTTAGATTGAAAGCCA | 54.3 | | 20 |
| 2. | *PSBB* | | | GCCGGAACTATGTGGTAT | 53.8 | 18 | GACCAAGCTTCTGATAAAC | 53.0 | | 19 |
|  | | Reference gene primer sequences used for quantitative RT-PCR | | | | | | |  |  |
| 1. | | | Actin | GACTGCCAAGACCAGCT | 54.9 | 17 | CTTCCTAATATCCACGTCG | 55.0 | | 19 |

| **S.No** | **Gene** | **Gene ID** |
| --- | --- | --- |
| 1. | *PSBA* | 803183 |
| 2. | *PSBB* | 803181 |


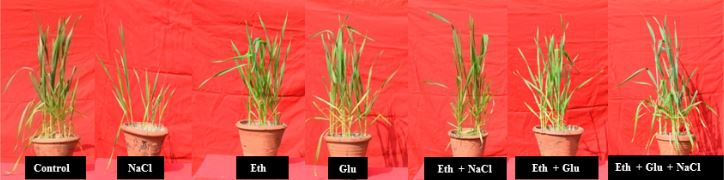


Figure S1.

**
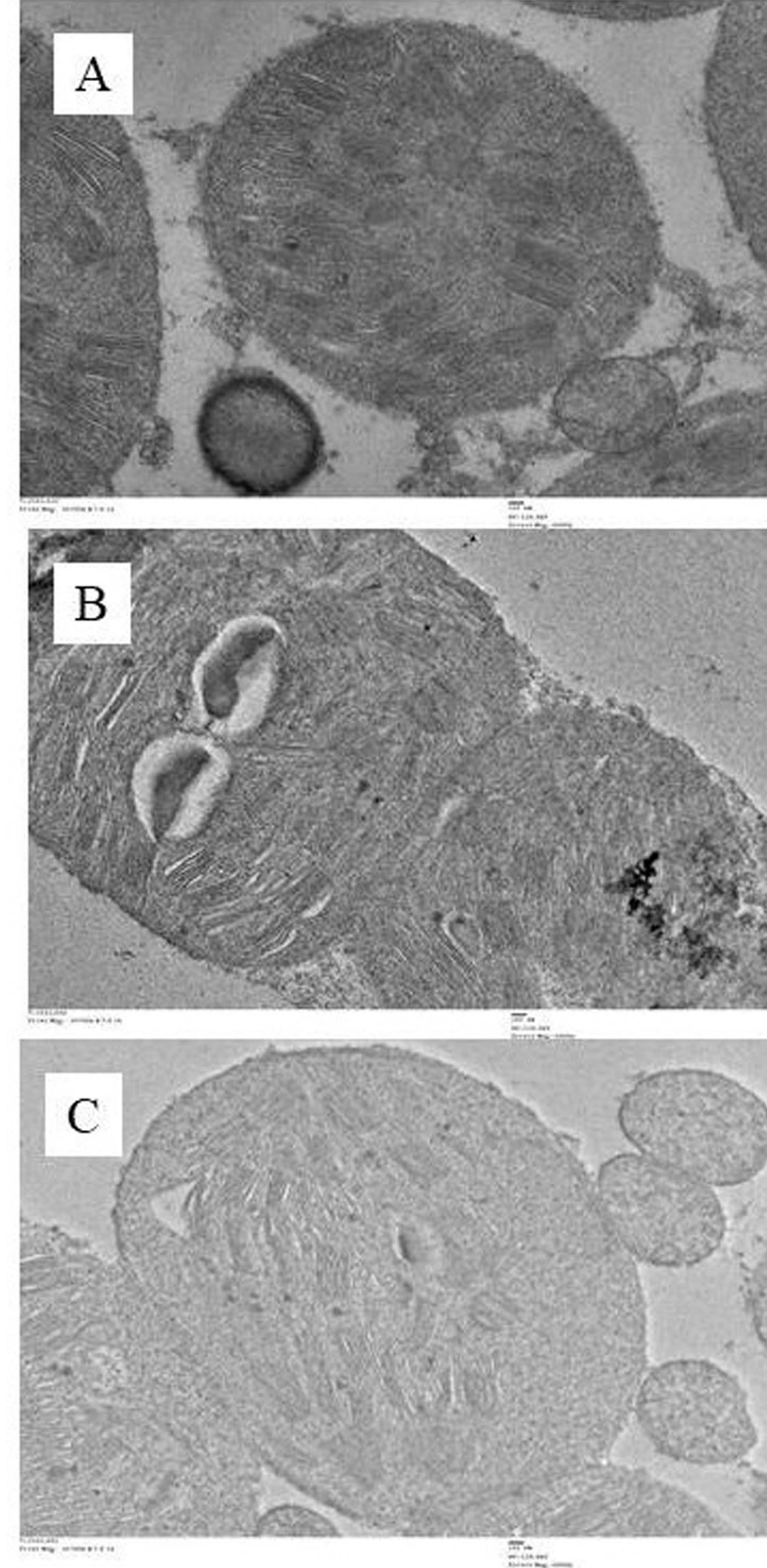
**

**Figure S2**

**Supplementary File S1. Methodology for the measurement of various parameters**

**Chlorophyll Fluorescence**

Minimal fluorescence (Fo) and maximum fluorescence (Fm) were measured in dark-adapted leaves with a low measuring beam at a light intensity of 125 mmol m^-2^ s^-1^, whereas under light-adapted condition, minimal fluorescence (Fo’) and maximum fluorescence (F_m_’) were measured in the same leaves with a saturating light intensity (720 mmol m^-2^ s^-1^) together with steady state fluorescence (F_s_). The variable fluorescence (F_v_ and F_v_^’^) was calculated using the values of F_m_ - F_o_ and F_m_^’^- F_o_^’^ and PSII was determined as F_m_^’^-F_s_^’^/ F_m_^’^, maximal efficiency of PSII by using F_v_/F_m_ and intrinsic efficiency of PS II by using F_v’_/F_m_’. Using the fluorescence parameters in both light and dark-adapted states, photochemical quenching (qP) and non-photochemical quenching (NPQ) were calculated. qP were calculated as F_m_^’^ – F_s_/ F_m_^’^ whereas NPQ was calculated as F_m_ – F_m_^’^/ F_m_^’^ (Maxwell and Johnson, 2000). Electron transport rate was calculated by using the following formula PSII × photosynthetic photon flux density × 0.5 × 0.84 as suggested by Krall and Edwards (1992).

**Contents of H_2_O_2_ and TBARS**

Leaf H_2_O_2_ was determined by adopting the method of Okuda et al. (1991). Details have described in the Supplemental File S1. The status of lipid peroxidation in leaves was estimated by the method described by Dhindsa et al. (1981) as the content of thiobarbituric acid reactive substances (TBARS). Leaf H_2_O_2_ was determined by adopting the method of Okuda et al. (1991).

**Assay of enzyme activities of ascorbate-glutathione pathway**

Fresh leaves (200 mg) were homogenized with an extraction buffer containing 0.05% (v/v) Triton X-100 and 1% (w/v) PVP in potassium-phosphate buffer (100 mM, pH 7.0) using chilled mortar and pestle. The homogenate was centrifuged at 15,000 x g for 20 min at 4^o^C. The supernatant obtained after centrifugation was used for the assay of SOD (EC; 1.15.1.1) and GR (EC; 1.6.4.2) enzymes. For the assay of APX (EC; 1.11.1.11), 2.0 mM ascorbate was supplemented with extraction buffer.

The activity of SOD was assayed by the method of Beyer and Fridovich (1987) and Giannopolitis and Ries (1977) through monitoring the inhibition of photochemical reduction of nitro blue tetrazolium (NBT). The activity of APX was determined following the method of Nakano and Asada (1981) by recording the decrease in the absorbance of ascorbate at 290 nm. The activity of GR was determined by the method of Foyer and Halliwell (1976) by monitoring the glutathione-dependent oxidation of NADPH at 340 nm.

**SOD activity**

The reaction mixture (5.0 ml) contained 5.0 mM HEPES buffer (pH 7.6), 0.1 mM EDTA, 50 mM Na_2_CO_3_ (pH 10.0), 13 mM methionine, 0.025% (v/v) Triton X-100, 63 µmol NBT,1.3 µmol riboflavin and the enzyme extract was illuminated for 15min (360 µmol m^-2^ s^-1^) and a control set was also illuminated for correcting the background absorbance. A unit of SOD was defined as the amount of enzyme required to cause 50% inhibition of the reaction of NBT at 560 nm. The activity of SOD was calculated by using the extinction coefficient 0.036 mM^-1^ cm^-1^. One unit of enzyme is the amount necessary to decompose 1.0 μmol of H_2_O_2_ per min at 25 ºC.

**APX activity**

The activity of APX was determined following the method of Nakano and Asada (1981) by recording the decrease in the absorbance of ascorbate at 290 nm. A 1.0 mL assay mixture contained 50 mM phosphate buffer (pH 7.0), 0.1 mM EDTA, 0.5 mM ascorbate and 0.1 mM H_2_O_2_, and the enzyme extract. APX activity was calculated by using the extinction coefficient of 2.8 mM^-1^cm^-1^. One unit of the enzyme is the amount necessary to decompose 1 µmol of substrate per min at 25^o^C.

**GR activity**

The activity of GR was determined by the method of Foyer and Halliwell (1976) by monitoring the glutathione-dependent oxidation of NADPH at 340 nm. The reaction mixture contained phosphate buffer (25 mM, pH 7.8), 0.5 mM GSSG, 0.2mM NADPH, and the enzyme extract. The activity of GR was calculated by using the extinction coefficient 6.2 mM^-1^ cm^-1^. One unit of enzyme is the amount necessary to decompose 1 µmol of NADPH per min at 25^o^C.

**GSH content**

Reduced glutathione was assayed by an enzyme recycling procedure in which it was sequentially oxidized by 5, 5-dithiobis-2-nitrobenzoic acid (DTNB) and reduced by NADPH in the presence of GR. For specific assay of GSSG, GSH was masked by derivatization with 2-vinylpyridine. Fresh leaf tissues (500 mg) were ground in liquid nitrogen using mortar and pestle and suspended in 2 ml of 5% (w:v) sulfosalicylic acid. The centrifugation was done at 12,000×g for 10 min. A 300 μL aliquot of supernatant was removed and neutralized by addition of 18 μL 7.5 M triethanolamine. To determine concentrations of GSH plus GSSG, one 150 μL sample was then used. Another sample was pre-treated with 3 μL 2-vinylpyridine for 60 min at 20ºC to mask the GSH by derivatization, to allow the subsequent determination of GSSG alone. In each case, 50 μL aliquots of the two types of sample were mixed with 700 μL 0.3 mM NADPH, 100 μL DTNB and 150 μl buffer containing 125 mM sodium phosphate, 6.3 mM EDTA (pH 6.5). A10 μL aliquot of GR (5 U ml^-1^) was then added and the change in absorbance at 412 nm monitored at 30ºC. Standard curve was prepared from GSH, covering a range of 5-55 nmol. For oxidized glutathione, standard curve covering a range of 1-5 nmol was used.

**Estimation of ethylene level**

Level of ethylene was estimated using gas chromatograph by cutting 500 mg of plant leaf into small pieces and placed into 30 mL tubes containing moist paper for minimizing the evaporation from the tissues and stoppered with secure rubber caps and kept in light for 2 h under the same condition used for the plant growth. An earlier experiment showed that 2 h of incubation time was appropriate for ethylene detection without the interference of wound induced ethylene, which started after 2 h of leaf incubation. A 1 mL of gas samples from the tubes were taken by a hypodermic syringe and assayed on a gas chromatograph (Nucon 5700, New Delhi, India) endowed with a 1.8 m Porapack N (80-100 mesh) column, a flame ionization detector and data station. Nitrogen was used as the carrier gas. The flow rates of hydrogen, nitrogen and oxygen were 30, 30 and 300 mL min^-1^, respectively; the detector was set at 150^o^C. Ethylene was detected based on retention time and measured by comparison with peaks from standard ethylene concentration.
